# Development of Enhanced Machine Learning Models for Predicting Type 2 Diabetes Mellitus Using Heart Rate Variability: A Retrospective Study

**DOI:** 10.7759/cureus.80933

**Published:** 2025-03-21

**Authors:** Vinni S Fengade, Hira Swati, Manoj Chandak, Rekha Rattan, Anish Singhal, Prathamesh Kamble, Mrunal Phatak, Nitin John

**Affiliations:** 1 Computer Science and Engineering, Shri Ramdeobaba College of Engineering and Management, Nagpur, IND; 2 Research and Innovations, Shri Ramdeobaba College of Engineering and Management, Nagpur, IND; 3 Physiology, All India Institute of Medical Sciences, Bibinagar, Bibinagar, IND; 4 Physiology, All India Institute of Medical Sciences, Nagpur, Nagpur, IND

**Keywords:** cardiac autonomic neuropathy, diabetes mellitus, heart rate variability, machine learning, non-invasive screening

## Abstract

Introduction: With its rising prevalence and serious complications, type 2 diabetes mellitus (T2DM) is a major worldwide health burden that calls for early detection using non-invasive screening techniques. Existing screening techniques, including OGTT, HbA1c, and fasting plasma glucose, have drawbacks in terms of accessibility, expense, and invasiveness. Recent developments in heart rate variability (HRV) analysis and machine learning (ML) offer a possible non-invasive substitute for diabetes screening. Previous research on HRV-based ML models in the classification of diabetes has issues with generalizability. The objective of this study is to develop and validate ML models using HRV features: time-domain, frequency-domain, and nonlinear HRV, to improve the prediction of T2DM. The study also evaluates the developed ML model's effectiveness against existing ML models.

Method: A retrospective dataset comprising 519 individuals (261 T2DM patients and 258 non-diabetic controls) was collected from the Autonomic Function Testing (AFT) laboratory repositories. To ensure comparability of age, gender, height, and weight among groups, post-hoc matching was used. HRV features were extracted from five-minute ECG recordings using the PowerLab data acquisition system and LabChart HRV module (ADInstruments, Sydney, Australia), following the European Society of Cardiology Task Force guidelines. An 80:20 train-test split was used to train and assess ML models, such as Logistic Regression, K-Nearest Neighbors (KNNs), Random Forest, Gradient Boosting, XGBoost, LightGBM, CatBoost, and AdaBoost. Accuracy, precision, recall, F1-score, area under the curve (AUC) for the receiver operating characteristic (ROC), sensitivity, and specificity were among the performance indicators. GridSearchCV was used for hyperparameter adjustment to maximize model performance.

Results: The baseline characteristics of the non-diabetic and T2DM groups were similar (p>0.05). HRV analysis showed substantial decreases in the diabetic group's time-domain (SDNN - SD of Normal-to-Normal Intervals/RMSSD - RMS of Successive Differences), frequency-domain (Low/High Frequency - LF/HF), and nonlinear (SD2 - SD of Poincaré Plot/CVRR - Coefficient of Variation of R-R Intervals) parameters (p<0.001). With a 91.2% accuracy rate and an AUC of 0.91, CatBoost outperformed other ML models in terms of prediction. LightGBM and Random Forest, which demonstrated high sensitivity and specificity, trailed closely behind. KNN achieved the highest accuracy (98.2%) and AUC (0.99), followed by Random Forest (96.4%) and CatBoost (94.5%), while hyperparameter modification further enhanced performance. CatBoost demonstrated the highest predictive performance, with an accuracy of 91.2% and an AUC of 0.91. According to correlation analysis, the most important HRV characteristics for diabetes prediction were SD2, SDRR (SD of R-R Intervals), and CVRR.

Conclusion: This study validates the utility of HRV-based ML models for non-invasive T2DM prediction, with ensemble models like CatBoost and LightGBM demonstrating superior performance when compared to the results of prior ML models. The optimized ML model, integrated with wearable medical technology for real-time monitoring, offers a scalable, affordable, and non-invasive alternative for diabetes screening. To improve generalizability and clinical use, future studies should investigate wearable-based HRV monitoring, multimodal AI models, and longitudinal validation.

## Introduction

Diabetes mellitus (DM) is a major global health concern, affecting 463 million people - 10% of the population - with projections reaching 12.8% by 2045 [1]. India, known as the "diabetes capital," accounts for 17% of global diabetics [2]. DM is a complex disorder with multiple systemic complications, such as cardiovascular diseases, neuropathy, and nephropathy, highlighting the need for early diagnosis. Current screening methods, including fasting plasma glucose, OGTT, and HbA1c, face challenges like invasiveness, cost, and inconvenience [3]. Point-of-care devices, like glucometers, though widely used, require painful pricking and costly test strips, limiting adoption. Non-invasive methods, like continuous glucose monitoring and optical glucose monitoring, show potential but remain costly and underdeveloped [4]. There is a pressing need for an affordable, non-invasive, rapid, and reliable screening tool with high specificity and good sensitivity for widespread use.

To address these challenges, machine learning (ML)-based screening approaches are promising alternatives, leveraging non-invasive tools like ECG, along with other parameters such as age, BMI, BP, sociodemographic, anthropometric, and biochemical data, and heart rate variability (HRV). ECG-based parameters, particularly HRV, offer a non-invasive, affordable solution for the early detection of diabetes-related changes. Additionally, with the integration of sensors like photoplethysmogram (PPG) in wearable devices, HRV can be continuously monitored, eliminating the need for ECG and enhancing the scalability of proposed HRV-based type 2 diabetes mellitus (T2DM) prediction [5]. Diabetes pathophysiology indicates that cardiovascular dysfunction arises early, with autonomic impairment preceding clinically noticeable dysglycemia [6]. Therefore, HRV can serve as a valuable parameter for early diabetes screening and prediction.

Several studies have explored the use of ECG-derived HRV parameters for diabetes prediction using ML models. One of the earliest studies exploring HRV alterations in diabetic patients was conducted by Faust et al. (2012), who conducted a study comparing HRV features between diabetic and healthy individuals, concluding that time-domain, frequency-domain, and nonlinear HRV parameters could be potential biomarkers for diabetes, though no ML modeling was applied [7]. Jian and Lim (2013) incorporated various ML classifiers, including Support Vector Machine (SVM), Gaussian Mixture Model (GMM), and Decision Trees (DTs), achieving an accuracy range of 52.76% to 79.93%, with the highest accuracy obtained using SVM with a Radial Basis Function (RBF) kernel [8]. Rajendra Acharya et al. (2013) analyzed nonlinear HRV parameters using classifiers like AdaBoost, DT, and SVM, reporting an overall accuracy of 90%, with sensitivity and specificity at 88.7% [9]. Acharya et al. (2015) extended their work using wavelet-based feature extraction methods and various ML classifiers, achieving a sensitivity of 92.02% and specificity of 91.46% [10]. Pachori et al. (2016) applied empirical mode decomposition for HRV feature extraction and used the least square-SVM (LS-SVM) classifier, obtaining the highest accuracy of 95.63% with the Morlet wavelet kernel function [11]. Swapna et al. (2018) explored deep learning models such as Convolutional Neural Networks (CNNs) and CNN-Long Short-Term Memory (CNN-LSTM), achieving an accuracy of 95.1%, with CNN performing better than CNN-LSTM [12]. Lastly, Aggarwal et al. (2020) conducted an animal study using artificial neural networks and SVM, reporting an accuracy of 86.3% and 90.5%, respectively [13]. These studies highlight the potential of HRV-based ECG parameters combined with ML models for non-invasive diabetes screening and also illustrate the progression in this field over time. The accuracy of classification has seen considerable enhancement over time, attributed to both advancements in ML technology and improved acquisition of ECG signals.

Despite promising findings, several limitations exist in the current literature on HRV-based diabetes screening. Faust et al. (2012) established a correlation between diabetes and HRV but lacked automated classification techniques, limiting its screening efficacy [7]. Additionally, many studies have focused on only one HRV domain - time, frequency, or nonlinear - despite HRV guidelines emphasizing the need for a comprehensive analysis of all domains for accurate interpretation [8-13]. Sample size constraints are another concern, as most studies involve only 15 to 25 diabetic subjects [7-13], reducing the generalizability of findings. Furthermore, ECG recordings used in these studies are often of very short durations, typically ranging from a few seconds to one minute [8-13], while HRV guidelines recommend a minimum duration of five minutes for reliable analysis. Another limitation is the broad age range (20-70 years) of diabetic participants in multiple studies, despite the known influence of aging on HRV parameters [8-11]. Addressing these limitations through larger sample sizes, longer ECG recordings, and comprehensive HRV analysis is crucial for improving the reliability and applicability of HRV-based diabetes screening.

In this context, we propose developing an advanced ML model utilizing HRV data from Autonomic Function Testing (AFT) laboratories at AIIMS. By leveraging a rich dataset and ML techniques, our goal is to enhance diabetes prediction accuracy, contributing to better screening strategies and improved patient outcomes. The objective of the present study is to develop and validate an ML algorithm for predicting T2DM using time, frequency, and nonlinear features extracted from HRV data sourced from the AFT laboratory repository at AIIMS and to compare its results with existing models.

## Materials and methods

Datasets

The present study was a collaborative effort between AIIMS Nagpur, AIIMS Bibinagar, and Shri Ramdeobaba College of Engineering and Management, Nagpur, India. HRV data from T2DM patients and healthy controls were collected at the AFT labs of both AIIMS institutions, while the engineering institute developed the ML model using these datasets. The collaboration included interdisciplinary discussions, ethical data sharing, and joint analysis to ensure the study's accuracy and robustness.

This study utilized retrospective data from both AIIMS AFT laboratories, comprising demographic details, clinical history, diagnosis, and raw ECG signals of both T2DM patients and non-diabetic individuals. The T2DM group was identified based on the American Diabetes Association criteria. We included patients aged 18 to 55 years without systemic comorbidities, as our retrospective dataset primarily comprised this age group. This range minimizes age-related HRV variability, which is particularly pronounced in individuals above 60 and below 18 years. The selection aligns with early to mid-life diabetes screening, where timely intervention is most effective. A total of 519 datasets were initially retrieved, consisting of 261 T2DM patients and 258 non-diabetic individuals. To ensure an appropriate comparison group, post-hoc matching was performed for the control group based on age, gender, height, and weight. Potential non-diabetic controls were identified from laboratory records, including individuals previously tested for HRV studies and those referred from outpatient departments. An initial set of 258 non-diabetic controls was selected; however, upon reevaluation, 22 individuals were excluded due to age mismatches or the presence of mild cardiovascular conditions, such as hypertension, dyslipidemia, or previous syncope episodes. These adjustments led to a final matched dataset of 261 T2DM patients and 244 non-diabetic controls. The post-hoc matching process involved frequency matching, ensuring similar distributions of age, gender, height, and weight between groups. Statistical validation was conducted using t-tests for continuous variables (age, height, weight, and BMI) and Chi-square tests for gender to confirm comparability between cases and controls.

For T2DM patients, we retrieved the duration of diabetes, fasting blood glucose (FBG), postprandial blood glucose (PPBG), and HbA1c levels closest to the date of HRV testing. However, since FBG, PPBG, and HbA1c were not routinely recorded for non-diabetic individuals, these values were unavailable. In this study, missing values for FBG, PPBG, or HbA1c were identified in 19 T2DM patients, although each of these patients had at least one of these values available. To ensure data completeness while minimizing bias, we employed K-Nearest Neighbors (KNN) imputation, an ML-based approach, using Python programming (Python Software Foundation, Wilmington, DE, USA). This method estimates missing values by identifying the five nearest data points (k = 5) based on available features, including age, gender, BMI, duration of diabetes, and available FBG/PPBG/HbA1c (if any), and then imputes the missing values using the weighted average of these neighbors. KNN imputation was preferred over mean or median imputation to preserve the natural variability in glucose levels and prevent distortion in the dataset. To validate the imputation, we compared the distribution of FBG and PPBG before and after imputation, ensuring that the imputed values aligned with overall data trends. This approach enabled the retention of all records for these 19 patients for subsequent analysis, although biochemical parameters were not included as features in the ML algorithm in this study for T2DM classification.

HRV data collection and feature extraction

HRV data were acquired using the PowerLab data acquisition system (ADInstruments, Sydney, Australia) and analyzed with the LabChart HRV module (ADInstruments, Sydney, Australia). ECG signals were recorded in Lead II configuration at a sampling frequency of 1000 Hz, ensuring high-resolution signal acquisition. Integrated signal conditioning with band-pass filtering (0.5-100 Hz) was applied to minimize motion artifacts and baseline drift. Before recording, participants were briefed on the procedure, and written informed consent was obtained. ECG was recorded in the supine position for 15 minutes, with the final five minutes used for HRV analysis. Data collection was conducted in a temperature-controlled laboratory (22°C-24°C) with minimal external disturbances. HRV analysis followed the Task Force guidelines of the European Society of Cardiology and the North American Society of Pacing and Electrophysiology [14], extracting time-domain (SDNN, or SD of Normal-to-Normal Intervals; RMSSD, or RMS of Successive Differences; NN50; pNN50), frequency-domain (LF, or Low Frequency; HF, or High Frequency; LF/HF ratio), and non-linear (Poincaré plot’s SD1, SD2, and SD1:SD2 ratio) parameters using the LabChart HRV module. Ectopic beats and artifacts were detected and corrected using the software’s automated artifact detection algorithm to ensure data reliability.

ML models

The dataset consisted of T2DM patients and healthy controls, making it a binary classification problem for ML modeling. To implement the ML model, we used Python-based data manipulation and ML libraries. As a first step, the dataset was split into 80% training and 20% testing sets to ensure proper model validation.

Several ML models were explored for classification. Logistic Regression, a generalized linear model (GLM), was employed to estimate the probabilities of a binary outcome using a logistic function. It assumes a linear relationship between input features and the log odds of the outcome, making it interpretable and computationally efficient. KNN was also evaluated, which predicts the class of a new data point based on the majority vote of its "k" closest neighbors, using distance metrics like Euclidean or Manhattan distances. The Random Forest Classifier, an ensemble learning method, was included in the study due to its robustness against overfitting, as it aggregates multiple DTs trained on bootstrapped samples with randomly selected features at each split. Additionally, the Gradient Boosting Classifier was tested, which builds DTs sequentially to correct errors of the previous trees and optimizes the loss function using gradient descent. XGBoost, an optimized form of gradient boosting that integrates regularization techniques and parallel computing for improved efficiency, was considered. LightGBM, developed by Microsoft (Microsoft® Corp., Redmond, WA, USA), was selected for speed and efficiency, particularly in handling large datasets through its gradient-based one-side sampling (GOSS) technique. CatBoost Classifier, designed by Yandex (Yandex LLC, Moscow, Russia), was also evaluated, as it efficiently processes categorical data using ordered boosting to minimize overfitting. Finally, the AdaBoost Classifier, an adaptive boosting technique that assigns higher weights to misclassified instances in each iteration, was tested for its effectiveness in improving weak classifiers.

Statistical evaluation

The performance of each ML model was evaluated using various classification metrics, including accuracy (ACC), precision, F1-score, area under the curve (AUC) for the receiver operating characteristic (ROC), sensitivity, and specificity. To further refine model performance, correlation analysis was conducted to determine the strength of the association between each feature and classification accuracy. Lastly, hyperparameter tuning was performed using the GridSearchCV method to optimize model performance by selecting the best combination of hyperparameters.

## Results

Table 1 depicts the demographic and baseline characteristics of individuals with and without DM. The results suggest that age, gender, and BMI were comparable between groups, with no statistically significant differences (age: p = 0.537, gender: p = 0.872, BMI: p = 0.724), ensuring effective matching. However, the mean duration of diabetes in the DM group was approximately 64.32 ± 10.32 months, indicating that most diabetic patients in the study had a similar duration of the disease. The mean FBG was 158.9 ± 31.5 mg/dL, PPBG was 257.5 ± 36.7 mg/dL, and HbA1c was 8.9 ± 1.2%, indicating a non-euglycemic state in the study population.

**Table 1:** Demographic and baseline parameters between individuals with and without type 2 diabetes mellitus (T2DM) p-values indicate statistical significance, with a 95% confidence interval (CI) representing the range of differences between groups.

Sr No	Baseline Parameters	T2DM Group (Mean ± SD)	Non-DM Group (Mean ± SD)	95% CI	p-value
1	Age (years)	32.82 ± 9.26	32.32 ± 9.21	-1.13 to 2.08	0.537
2	Gender (Male, %)	45.77%	48.08%	-	0.872
2	Gender (Female, %)	54.23%	46.15%	-	0.87
3	BMI (kg/m²)	24.32 ± 2.35	24.39 ± 2.40	-0.51 to 0.33	0.724
4	Diabetes Duration (months)	64.34 ± 10.32	-	-	-
5	Fasting Blood Glucose (mg/dL)	158.9 ± 31.5	-	-	-
6	Postprandial Blood Glucose (mg/dL)	257.5 ± 36.7	-	-	-
7	HbA1c (%)	8.9 ± 1.2	-	-	-

To evaluate further, Table 2 shows the comparison of HRV parameters in the diabetic and non-diabetic groups. The DM group showed significantly lower HRV metrics, including average RR, SDRR, RMSSD, total power, and very low frequency (VLF) power (p < 0.001 for all). Sympathetic dominance was observed in the DM group, with a higher LF/HF ratio. Most HRV parameters showed significant differences, except for LF Power% (p = 0.328). These results indicate reduced autonomic function and increased sympathetic activity in individuals with T2DM.

**Table 2:** Comparison of heart rate variability (HRV) parameters between individuals with and without type 2 diabetes mellitus (T2DM) p-values indicate statistical significance, with a 95% confidence interval (CI) representing the range of differences between groups. Significant differences (p < 0.001) are represented by bold p-values. RR: R-R Interval; SDRR: Standard Deviation of RR Interval; CVRR: Coefficient of Variation of RR Interval; SDSD: Standard Deviation of Successive Differences; RMSSD: Root Mean Square of Successive Differences; pRRx (%): Percentage of RR Intervals Greater than x; SD1: Standard Deviation 1; SD2: Standard Deviation 2; VLF: Very Low Frequency; LF: Low Frequency; HF: High Frequency

Sr No	HRV Parameters	T2DM Group (Mean ± SD)	Non-DM Group (Mean ± SD)	95% CI	p-value
1	Average RR (ms)	753.29 ± 117.7	819.61 ± 134.91	-96.36 to -36.32	<0.001
2	SDRR (ms)	22.75 ± 10.65	62.43 ± 23.49	-43.71 to -37.18	<0.001
3	CVRR	0.0298 ± 0.0127	0.0771 ± 0.0345	-0.05 to -0.04	<0.001
4	SDSD (ms)	17.55 ± 15.63	54.99 ± 29.88	-47.43 to -37.44	<0.001
5	RMSSD (ms)	17.61 ± 16.15	54.94 ± 29.85	-43.11 to -37.32	<0.001
6	pRRx (%)	2.97 ± 2.98	27.68 ± 19.33	-26.71 to -21.83	<0.001
7	SD1 (ms)	12.42 ± 11.05	38.89 ± 21.29	-27.55 to -21.67	<0.001
8	SD2 (ms)	29.48 ± 13.67	74.8 ± 25.75	-49.72 to -38.52	<0.001
9	Total Power (ms²)	520.05 ± 451.3	3969.77 ± 3068.77	-3968.53 to -3449.72	<0.001
10	VLF Power (ms²)	244.05 ± 245.27	1369.43 ± 1165.63	-1125.39 to -917.88	<0.001
11	VLF Power (%)	48.48 ± 18.29	37.03 ± 16.6	7.27 to 13.36	<0.001
12	LF Power (ms²)	144.16 ± 172.34	1032.3 ± 872.94	-1165.08 to -721.44	<0.001
13	LF Power (%)	27.15 ± 13.33	26.55 ± 9.93	-0.82 to 2.44	0.328
14	LF Power (nu)	52.05 ± 30.61	24.8 ± 18.47	23.01 to 32.94	<0.001
15	HF Power (ms²)	79.79 ± 165.64	1497.45 ± 1653.07	-1730.65 to -1005.96	<0.001
16	HF Power (%)	23.39 ± 15.95	37.97 ± 16.79	-17.63 to -11.53	<0.001
17	HF Power (nu)	43.18 ± 29.28	63.04 ± 17.78	-23.65 to -14.91	<0.001

The performance of several ML models in predicting T2DM using HRV parameters was evaluated based on key classification metrics, including accuracy, precision, F1 score, ROC AUC, sensitivity, and specificity (Table 3). Among the models tested, CatBoost demonstrated the highest predictive performance, achieving the best accuracy, precision, and ROC AUC, while maintaining strong sensitivity and specificity, making it the most reliable model for T2DM classification. LightGBM followed closely, exhibiting high accuracy and the highest specificity, indicating its effectiveness in correctly identifying non-diabetic individuals. Gradient Boosting Machine (GBM) and Random Forest also showed competitive performance, with GBM maintaining a balanced sensitivity and specificity, while Random Forest achieved the highest sensitivity, suggesting its strength in detecting diabetic cases. Logistic Regression and XGBoost performed well, providing consistent accuracy and a balanced trade-off between precision and sensitivity, making them viable options for classification. KNN exhibited moderate performance, though with slightly lower sensitivity, indicating potential limitations in capturing all diabetic cases. AdaBoost had the lowest accuracy among the models, but still maintained a balanced trade-off between precision and sensitivity, suggesting its applicability in specific scenarios. Overall, boosting algorithms, particularly CatBoost, demonstrated superior predictive capability for T2DM detection using HRV parameters. Other ensemble models, such as LightGBM, also performed competitively, while Logistic Regression provided an interpretable alternative with reasonable classification accuracy. These findings highlight the effectiveness of boosting algorithms in predictive modeling for T2DM, with CatBoost emerging as the most robust model.

**Table 3:** Performance metrics of AI models for predicting type 2 diabetes mellitus (T2DM) using heart rate variability (HRV) parameters AUC: Area Under Curve; ROC: Receiver Operating Characteristic

Models	Accuracy	Precision	F1 Score	ROC AUC	Sensitivity	Specificity
Logistic Regression	0.8836	0.8793	0.8717	0.8820	0.8653	0.8987
KNN	0.8655	0.8673	0.8509	0.8633	0.8355	0.8912
Random Forest	0.8909	0.8705	0.8837	0.8910	0.8979	0.8909
GBM	0.8945	0.8810	0.8853	0.8941	0.8901	0.8981
AdaBoost	0.8545	0.8368	0.8466	0.8525	0.8576	0.8475
XGBoost	0.8800	0.8689	0.8720	0.8791	0.8753	0.8830
LightGBM	0.9018	0.8975	0.8939	0.9010	0.8907	0.9112
CatBoost	0.9127	0.9064	0.9059	0.9124	0.9062	0.9186

The overall predictive performance of the models is further supported by the confusion matrix analysis (Table 4). The key findings are CatBoost achieved the highest true positives (22.8) and true negatives (27.4), indicating it is the most effective model in identifying both diabetic and non-diabetic individuals. Additionally, it recorded the lowest false positives (2.4) and false negatives (2.4), confirming its superior accuracy and precision. LightGBM also showed strong performance, while AdaBoost had relatively weaker outcomes. These results suggest that boosting algorithms, particularly CatBoost, are highly effective for the predictive modeling of T2DM using HRV parameters.

**Table 4:** Confusion matrix analysis of AI models for predicting type 2 diabetes mellitus (T2DM) using heart rate variability (HRV) parameters KNN: K-Nearest Neighbors; GBM: Gradient Boosting Machine

Models	True Positives	True Negatives	False Positives	False Negatives
Logistic Regression	21.8	26.8	3	3.4
KNN	21	26.6	3.2	4.2
Random Forest	22.6	26.4	3.4	2.6
GBM	22.4	26.8	3	2.8
AdaBoost	21.6	25.4	4.4	3.6
XGBoost	22	26.4	3.4	3.2
LightGBM	22.4	27.2	2.6	2.8
CatBoost	22.8	27.4	2.4	2.4

**Figure 1:**
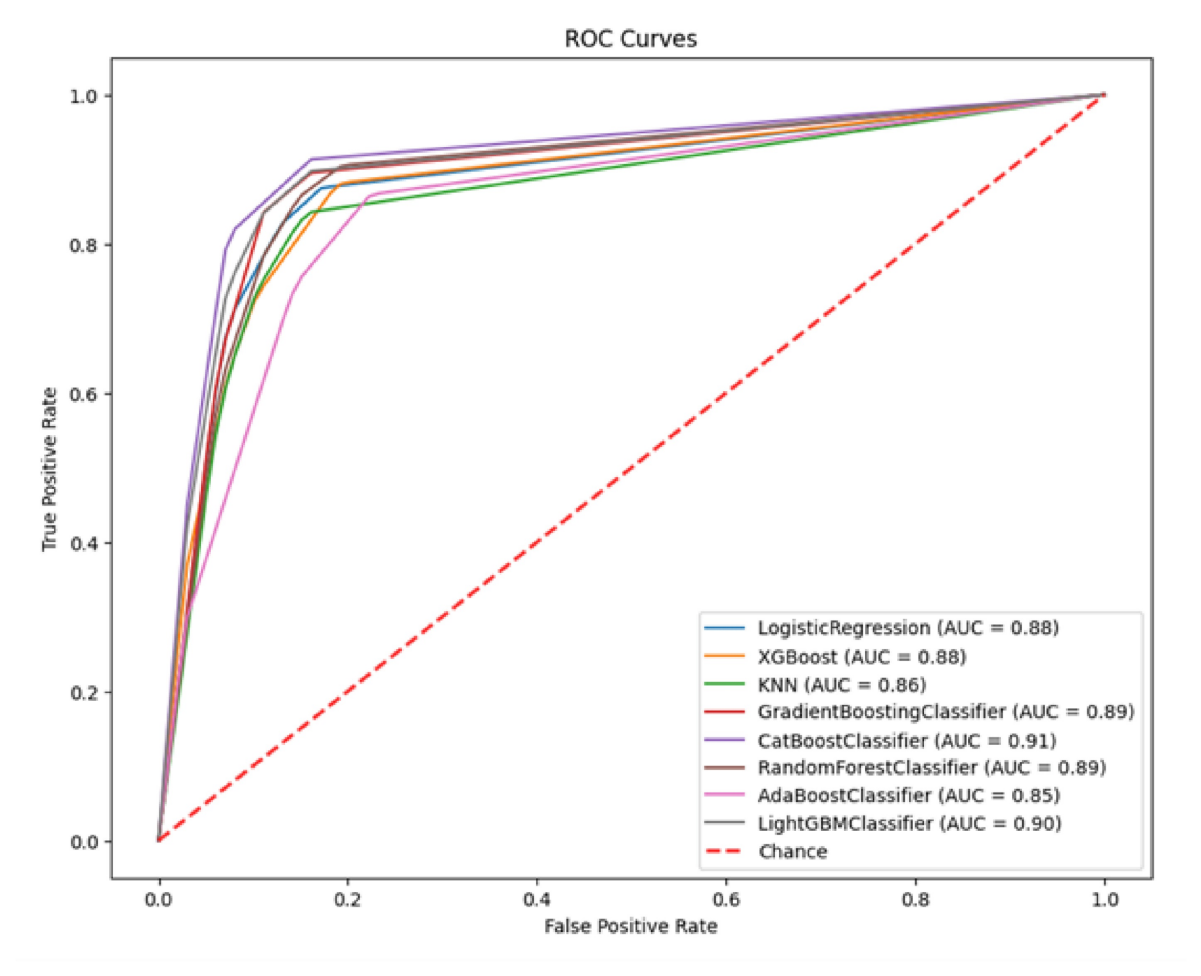
Receiver operating characteristic (ROC) curves comparing AI model performance in type 2 diabetes mellitus (T2DM) prediction using heart rate variability (HRV) parameters The receiver operating characteristic (ROC) curve illustrates the performance of various machine learning models in predicting type 2 diabetes mellitus (T2DM) based on heart rate variability (HRV) parameters. The area under the curve (AUC) represents the model’s ability to distinguish between diabetic and non-diabetic cases, with higher values indicating better discrimination. The diagonal red dashed line represents a random classifier (AUC = 0.50). KNN: K-Nearest Neighbors; GBM: Gradient Boosting Machine

To complement the confusion matrix analysis, Figure 1 presents the ROC curves, offering a visual representation of each model’s discriminative power based on HRV parameters. The AUC values demonstrate how well each model balances sensitivity and specificity, with higher AUC values indicating better classification performance. CatBoost achieved the highest AUC (0.91), confirming its superior predictive capability, followed closely by LightGBM (0.90) and Gradient Boosting (0.89). These results further validate that boosting algorithms, particularly CatBoost and LightGBM, outperform traditional models like Logistic Regression in T2DM prediction. 

The correlation analysis presented in Table 5 further elucidates the relationship between HRV parameters and the presence of T2DM, providing valuable insights into the most influential features for diabetes classification. While the ROC curves in Figure 1 demonstrated the overall predictive power of different ML models, the correlation findings help identify which HRV parameters contribute most to distinguishing diabetic individuals. The results indicate that time-domain and non-linear features exhibit the strongest association with diabetes, reinforcing their importance in classification. Conversely, frequency-domain metrics, including the LF/HF ratio and LF Power%, show weaker correlations, suggesting that time-domain and non-linear features of HRV are more predictive of T2DM than spectral components. This analysis supports the selection of HRV parameters that enhance model performance, further validating the effectiveness of ML-based approaches in diabetes prediction.

**Table 5:** Association of heart rate variability (HRV) features with diabetes in decreasing order of Pearson’s correlation coefficient p-values indicate statistical significance, with a Pearson correlation coefficient (r). Significant differences (p < 0.05) are represented by bold p-values. RR: R-R Interval; SDRR: Standard Deviation of RR Interval; CVRR: Coefficient of Variation of RR Interval; SDSD: Standard Deviation of Successive Differences; RMSSD: Root Mean Square of Successive Differences; pRRx (%): Percentage of RR Intervals Greater than x; SD1: Standard Deviation 1; SD2: Standard Deviation 2; VLF: Very Low Frequency; LF: Low Frequency; HF: High Frequency

HRV Parameters	Pearson Correlation Coefficient (r)	p-value
Average RR (ms)	-0.57	<0.001
SDRR (ms)	0.43	0.002
CVRR	0.39	0.005
SDSD (ms)	0.62	<0.001
RMSSD (ms)	0.61	<0.001
pRRx (%)	0.58	<0.001
SD1 (ms)	0.6	<0.001
SD2 (ms)	0.55	0.001
Total Power (ms²)	0.59	<0.001
VLF Power (ms²)	0.57	<0.001
VLF Power (%)	-0.31	0.051
LF Power (ms²)	0.5	<0.001
LF Power (%)	0.11	0.402
LF Power (nu)	-0.45	0.003
HF Power (ms²)	0.49	0.001
HF Power (%)	-0.28	0.098
HF Power (nu)	0.44	0.004

Building on the correlation analysis, which identified key HRV features associated with T2DM, Table 6 presents a comparative evaluation of ML models trained on these impactful features. This analysis provides deeper insights into how different feature sets influence model performance across key metrics such as accuracy, precision, sensitivity, F1 score, and ROC AUC. Notably, the Random Forest Classifier emerged as the top-performing model, achieving the highest average accuracy and sensitivity, highlighting its effectiveness in detecting both diabetic and non-diabetic individuals. Other ensemble models, including LightGBM and CatBoost, also demonstrated strong predictive power, leveraging diverse HRV feature sets to achieve high accuracy and balanced sensitivity-specificity trade-offs. While KNN exhibited the highest precision, it had lower sensitivity, indicating a potential limitation in detecting all diabetic cases. XGBoost, Logistic Regression, and Gradient Boosting Classifiers showed balanced performance, making them viable alternatives, while AdaBoost had the lowest accuracy, suggesting it is less effective compared to other models. Overall, the findings reaffirm that ensemble-based approaches, particularly Random Forest, LightGBM, and CatBoost, offer superior predictive performance for T2DM detection using HRV parameters. The varying contributions of HRV features across models emphasize the importance of selecting optimal feature sets for improved classification accuracy. These results further strengthen the case for ML-based approaches in clinical decision support for diabetes detection.

**Table 6:** Performance of AI models with key features impacting type 2 diabetes mellitus (T2DM) prediction KNN: K-Nearest Neighbors; GBM: Gradient Boosting Machine; HRV: Heart Rate Variability; ROC: Receiver Operating Characteristic; AUC: Area Under the Curve

Model	Impacting HRV Features	Average Accuracy	Average Precision	Average Sensitivity	Average F1 Score	Average ROC AUC Score
Random Forest Classifier	I - XIII	0.9091	0.8927	0.9033	0.8975	0.9084
LightGBM Classifier	I - XIV	0.9018	0.9084	0.8711	0.8891	0.8980
CatBoost Classifier	I - VI	0.9018	0.9154	0.8637	0.8884	0.8979
KNN	I - VIII	0.8945	0.9301	0.8320	0.8781	0.8882
XGBoost	I - XV	0.8945	0.8936	0.8711	0.8817	0.8916
Logistic Regression	I - XV	0.8909	0.9002	0.8589	0.8778	0.8895
Gradient Boosting Classifier	I - XII	0.8909	0.8939	0.8642	0.8782	0.8879
AdaBoost Classifier	I - XVI	0.8836	0.8788	0.8611	0.8680	0.8804

Hyperparameter tuning was performed to optimize the performance of ML models in T2DM prediction (Table 7).

**Table 7:** Hyperparameter tuning details for machine learning models used in diabetes mellitus prediction KNN: K-Nearest Neighbors; GBM: Gradient Boosting Machine

AI Model	Details of Parameters Used for Hyperparameter Tuning
Logistic Regression	{'C': 0.001, 'max_iter': 1000, 'solver': 'sag'}
KNN Classifier	{'metric': 'euclidean', 'n_neighbors': 7, 'weights': 'distance'}
Random Forest Classifier	{'max_depth': None, 'min_samples_leaf': 1, 'min_samples_split': 10, 'n_estimators': 150}
Gradient Boosting Classifier	{'learning_rate': 0.01, 'max_depth': 3, 'n_estimators': 150}
AdaBoost Classifier	{'learning_rate': 0.01, 'n_estimators': 50}
XGBoost Classifier	{'gamma': 0.2, 'learning_rate': 0.01, 'max_depth': 5, 'n_estimators': 50}
LightGBM Classifier	{'learning_rate': 0.01, 'max_depth': -1, 'n_estimators': 50}
CatBoost Classifier	{'depth': 3, 'iterations': 100, 'learning_rate': 0.01}

Among them, Random Forest achieved the highest overall accuracy and sensitivity, making it the most effective in distinguishing diabetic and non-diabetic individuals (Table 8). KNN demonstrated the highest precision but had lower sensitivity, while LightGBM and CatBoost maintained strong classification performance with a balanced trade-off between sensitivity and specificity. XGBoost, Gradient Boosting, and Logistic Regression exhibited consistent results, whereas AdaBoost showed the lowest sensitivity, limiting its ability to detect all diabetic cases.

**Table 8:** Performance metrics of machine learning models after hyperparameter tuning for predicting diabetes mellitus using heart rate variability (HRV) data KNN: K-Nearest Neighbors; GBM: Gradient Boosting Machine; AUC: Area Under the Curve

Model	Accuracy	AUC Score	Sensitivity	Specificity	Precision	F-Score
Logistic Regression	0.945	0.9905	0.870	1.000	1.000	0.930
KNN Classifier	0.982	0.9918	0.957	1.000	1.000	0.978
Random Forest Classifier	0.964	0.9973	0.957	0.969	0.957	0.957
Gradient Boosting Classifier	0.945	0.9864	0.913	0.969	0.955	0.933
AdaBoost Classifier	0.927	0.9946	0.826	1.000	1.000	0.905
XGBoost Classifier	0.945	0.9905	0.913	0.969	0.955	0.933
LightGBM Classifier	0.945	0.9918	0.870	1.000	1.000	0.930
CatBoost Classifier	0.945	0.9959	0.913	0.969	0.955	0.933

**Figure 2:**
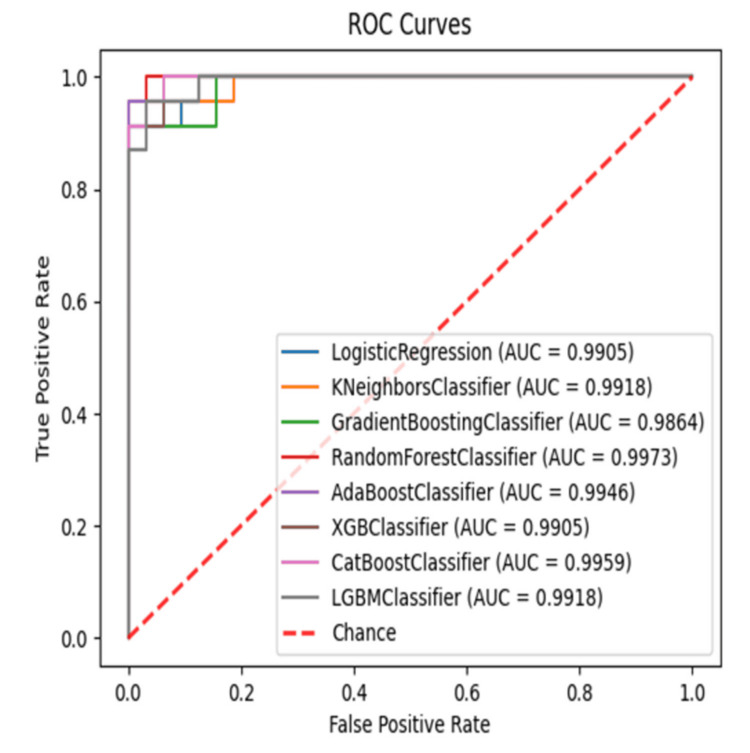
Receiver operating characteristic (ROC) curves comparing AI model performance after hyperparameter tuning for predicting type 2 diabetes mellitus (T2DM) using heart rate variability (HRV) parameters The receiver operating characteristic (ROC) curve illustrates the performance of various machine learning models in predicting type 2 diabetes mellitus (T2DM) based on heart rate variability (HRV) parameters after hypertuning. The area under the curve (AUC) represents the model’s ability to distinguish between diabetic and non-diabetic cases, with higher values indicating better discrimination. The diagonal red dashed line represents a random classifier (AUC = 0.50). KNN: K-Nearest Neighbors; GBM: Gradient Boosting Machine

The comparison of AI model performance before (Figure 1) and after tuning (Figure 2) reveals significant improvements across key metrics. KNN and Random Forest showed the highest gains, with accuracy increasing to 0.982 and 0.964, respectively, and AUC scores rising to 0.9918 and 0.9973. Sensitivity improved notably, especially in KNN (0.8355 to 0.957) and CatBoost (0.9062 to 0.913), while KNN achieved perfect specificity (1.000). Precision and F1 scores also increased, with KNN reaching 1.000 precision, and Random Forest improving its F1 score from 0.8837 to 0.957.

While most models benefited from tuning, AdaBoost showed a slight decline in precision and F1 score, indicating room for further optimization. Overall, hyperparameter tuning significantly enhanced model performance, with KNN emerging as the best classifier, reinforcing the importance of parameter optimization in improving predictive accuracy for T2DM detection.

## Discussion

In the present study, the demographic analysis confirmed that age, gender, and BMI were comparable between T2DM and non-diabetic groups (p > 0.05), ensuring effective matching. However, the diabetic group exhibited prolonged disease duration and a non-euglycemic state, with elevated FBG, PPBG, and HbA1c, reflecting chronic hyperglycemia. These findings align with established T2DM characteristics and suggest a potential link between prolonged hyperglycemia and autonomic dysfunction, warranting further investigation through HRV analysis. The study confirms reduced autonomic function and increased sympathetic activity in T2DM patients, reinforcing HRV as a reliable non-invasive biomarker for diabetes-related autonomic dysfunction. Several studies align with these observations [1,2,4].

This study highlights a strong correlation between HRV features and diabetes, with SD2 (ms), SDRR, or SD of R-R Intervals (ms), and CVRR, or Coefficient of Variation of R-R Intervals, emerging as key predictors. These findings reinforce the link between autonomic dysfunction and diabetes pathogenesis, supporting HRV as a potential biomarker for metabolic disorders. The high correlation of SD2 (ms) and SDRR (ms) with diabetes risk aligns with prior research [8,11,13], emphasizing nonlinear HRV's major parameters for prediction models of T2DM.

The results of our study provide a detailed comparison of various ML models trained on HRV data for predicting DM. Among the models, CatBoost stood out as the top performer. This model demonstrated its superior discriminative ability, as reflected by its AUC of 0.91. LightGBM and Random Forest also performed competitively and provided high precision and sensitivity values, which indicate their robustness in handling the complexity of HRV features for diabetes prediction. The success of these models highlights the importance of using ensemble techniques in complex clinical predictions, particularly when data dimensions and feature interactions are key considerations. Interestingly, models like Logistic Regression, while generally performing well, trailed behind the more advanced ensemble models. Logistic Regression's inability to capture nonlinear relationships likely contributed to its comparatively lower performance. This observation emphasizes the limitation of linear models in handling intricate biological data, such as HRV, where non-linear patterns may play a significant role in disease prediction. Table 9 compares the results of our study with previous research on HRV features for predicting T2DM using various ML models. Our findings reaffirm that tree-based ensemble models, such as Random Forest, Gradient Boosting, and CatBoost, consistently outperform other approaches in HRV-based T2DM prediction, leveraging their ability to capture nonlinear relationships and complex feature interactions effectively.

**Table 9:** Overview of studies on heart rate variability (HRV) features for predicting diabetes mellitus using various machine learning models FC: Fuzzy Classifier; GMM: Gaussian Mixture Model; ANN: Artificial Neural Network; SVM: Support Vector Machine; PNN: Probabilistic Neural Network; NBC: Naive Bayes Classifier; KNN: K-Nearest Neighbor; DM: Diabetes Mellitus; DT: Decision Tree Classifier; FSC: Fuzzy Sugeno Classifier; LS-SVM: Least Square-Support Vector Machine Classifier; RBF: Radial Basis Function; CNN: Convolutional Neural Network; LSTM: Long Short-Term Memory; GBM: Gradient Boosting Machine

Author	ML Models Used	Results in Brief Highest Accuracy (Model)
Faust et al. (2012) [7]	No ML modeling (Student's t-test)	Generated hypothesis that TD, FD, and NL parameters can predict DM
Jian and Lim (2013) [8]	FC, GMM, SVM, PNN, NBC, KNN, DT	79.93% (SVM with RBF kernel)
Rajendra Acharya et al. (2013) [9]	AdaBoost, DT, FSC, KNN, PNN, SVM	90% (AdaBoost)
Acharya et al. (2015) [10]	DT, KNN, NBC, SVM	92.02%
Pachori et al. (2016) [11]	LS-SVM (RBF, Morlet wavelet, Mexican kernel)	95.63% (Morlet wavelet kernel)
Swapna et al. (2018) [12]	CNN, CNN-LSTM	95.1%
Aggarwal et al. (2020) [13]	ANN, SVM	90.5% (SVM)
Present Study	Logistic Regression, KNN, Random Forest, Gradient Boosting, XGBoost, LightGBM, CatBoost, AdaBoost	98.2% (KNN) 96.4% (Random Forest) CatBoost, LightGBM, and Gradient Boosting performed well.

Our study also demonstrates the importance of hyperparameter tuning in enhancing model performance. By employing GridSearchCV, we were able to optimize the Logistic Regression model, achieving a significantly improved accuracy of 94.54%, along with a perfect precision score of 100%, and an AUC of 0.99. This highlights how even relatively simple models can be fine-tuned to achieve high performance with careful selection of hyperparameters, such as the regularization parameter "C" and solver type. Hyperparameter tuning plays a crucial role in improving model generalization and preventing overfitting, particularly when dealing with clinical data, where the stakes of false positives and false negatives are high. In this context, the remarkable performance of the tuned Logistic Regression model further validates its practical utility, particularly in scenarios where interpretability and simplicity are preferred over complexity.

The confusion matrix analysis provided additional insights into the classification capabilities of the models. CatBoost, with the highest number of true positives and true negatives, minimized both false positives and false negatives, making it highly reliable for clinical decision-making. In contrast, models like AdaBoost showed relatively higher false positives and false negatives, indicating potential trade-offs between sensitivity and specificity. From a clinical perspective, reducing false negatives is critical in diabetes prediction, as missing a diagnosis could delay intervention and worsen patient outcomes. Therefore, models like CatBoost and LightGBM, which balanced both sensitivity and specificity well, are ideal for clinical applications where early detection is crucial.

The ability of ML models to predict DM from HRV data has important clinical implications, as HRV is a non-invasive, readily obtainable biomarker that can provide valuable insights into a patient's metabolic health and autonomic function. The high accuracy of models like CatBoost and Random Forest in predicting diabetes from HRV features opens possibilities for integrating these models into routine screening programs, potentially aiding in the early detection of diabetes in at-risk populations. However, for DM, a diagnostic test should not only be rapid, non-invasive, affordable, specific, and sensitive, but it should also adopt a multi-system, holistic, and multifaceted approach, addressing both the subclinical stages of the disease and its complications, including potential metabolic dysregulation. The generalizability of these models must be evaluated further across diverse populations and larger datasets. Additionally, incorporating other physiological markers, such as glucose levels or lipid profiles, alongside HRV, could further enhance predictive accuracy and clinical utility. If HRV is established as a reliable predictive marker, it could serve not only as an indicator of DM but also as a gold standard for assessing cardiovascular health, including central nervous system autonomic activity and neuropathy.

A key strength of this study is its comprehensive HRV analysis, which integrates time-domain, frequency-domain, and nonlinear HRV features to provide a holistic assessment of autonomic dysfunction in diabetes. Unlike previous studies that focused on a single HRV domain, this approach ensures a more robust and nuanced evaluation of autonomic regulation. Additionally, the study employs a diverse range of ML models, including ensemble-based classifiers such as Random Forest, CatBoost, and LightGBM, which have demonstrated high predictive accuracy and reliability. Furthermore, the analysis is strengthened using a relatively large dataset (261 T2DM patients and 244 non-diabetic controls), with post-hoc matching based on demographic and anthropometric parameters, ensuring balanced group comparisons and minimizing confounding variables. These methodological strengths enhance the clinical relevance and potential applicability of HRV-based ML models for early diabetes detection and risk stratification.

Despite its strengths, this study has certain limitations that should be acknowledged. First, the cross-sectional design prevents the assessment of causal relationships between HRV alterations and T2DM progression, limiting insights into longitudinal changes in autonomic dysfunction. Additionally, the dataset was sourced from a specific clinical population at two study sites, which may restrict the generalizability of findings to broader or more diverse populations. While rigorous hyperparameter tuning was performed, the potential for overfitting in complex ensemble models such as Random Forest and CatBoost remains a concern, particularly when dealing with high-dimensional HRV features. Moreover, the study excluded patients with systemic comorbidities, which, while ensuring data purity, limits understanding of HRV variations in T2DM patients with coexisting conditions, like hypertension or cardiovascular disease.

Further validation in larger, multi-center cohorts with diverse ethnic and demographic backgrounds is needed to enhance the real-world applicability of HRV-based diabetes prediction models. Future research should focus on longitudinal studies, integrating wearable HRV monitoring, and assessing HRV’s predictive value in diabetes, prediabetes, impaired fasting glucose, and impaired glucose tolerance screening. Additionally, analyzing the relationship between HRV parameters and HbA1c across glycemic states could help track progressive autonomic dysfunction, with longitudinal HbA1c monitoring further enhancing early diabetes detection and refining predictive models.

In this study, CatBoost and LightGBM demonstrated high predictive performance, but combining multiple models in a hybrid or stacked ensemble strategy may improve robustness and generalizability, warranting further research.

Our study findings highlight the potential of HRV-based ML models as a non-invasive, cost-effective screening tool for early T2DM detection, particularly in asymptomatic individuals. HRV can be measured using wearable devices (e.g., smartwatches, fitness trackers, and portable ECG monitors), enabling real-time risk assessment without the need for laboratory-based blood tests. This approach could support remote monitoring and preventive healthcare strategies, especially in telemedicine and rural health programs. However, as this concept is still in its early stages, a large, multicentric study is required to validate its effectiveness as a screening tool.

## Conclusions

This study demonstrates the potential of HRV-based ML models as a non-invasive tool for predicting T2DM. By integrating time-domain, frequency-domain, and nonlinear HRV features, the study identifies SD2, SDRR, and CVRR as strong predictors of diabetes, reinforcing HRV’s role in assessing autonomic dysfunction. Among the models tested, Random Forest, LightGBM, and CatBoost showed superior predictive performance, with KNN emerging as the best-performing classifier (accuracy: 0.982 and AUC: 0.9918) after hyperparameter tuning. Comparing the results of our study with previous research on HRV features for predicting T2DM using various ML models, our findings reaffirm that tree-based ensemble models, such as Random Forest, Gradient Boosting, and CatBoost, consistently outperform other approaches in HRV-based T2DM prediction by effectively capturing nonlinear relationships and complex feature interactions. These findings highlight the clinical utility of AI-driven HRV analysis for early diabetes detection and risk stratification. However, limitations such as the cross-sectional design and dataset specificity call for longitudinal validation and external testing across diverse populations. Future research should focus on wearable-based HRV monitoring and multimodal AI models to enhance real-world applicability and improve preventive healthcare strategies.

## Appendices

Comparison of the best-performing CatBoost with other AI models for performance metrics in T2DM prediction: We compared the best-performing CatBoost model with various AI models for predicting T2DM using HRV parameters (Table 10). Bootstrap confidence intervals (95% CI) were calculated for differences in accuracy, precision, and AUC between each model and the best-performing CatBoost model. The results indicate that CatBoost consistently outperforms other models, with statistically significant improvements in key performance metrics, as reflected in the confidence intervals.

**Table 10:** Comparison of best-performing CatBoost with other AI models for performance metrics in T2DM prediction using bootstrap confidence intervals (95% CI) Bootstrap confidence intervals (95% CI) were calculated for differences in accuracy, precision, and AUC between each model and CatBoost. KNN: K-Nearest Neighbors; GBM: Gradient Boosting Machine; ROC: Receiver Operating Characteristic; AUC: Area Under the Curve

Model	Metric	Mean Difference	95% CI
Logistic Regression	Accuracy	0.03	(0.03, 0.03)
KNN	Accuracy	0.05	(0.04, 0.05)
Random Forest	Accuracy	0.02	(0.02, 0.02)
GBM	Accuracy	0.02	(0.02, 0.02)
AdaBoost	Accuracy	0.06	(0.06, 0.06)
XGBoost	Accuracy	0.03	(0.03, 0.04)
LightBoost	Accuracy	0.01	(0.01, 0.01)
Logistic Regression	Precision	0.03	(0.02, 0.03)
KNN	Precision	0.04	(0.04, 0.04)
Random Forest	Precision	0.04	(0.03, 0.04)
GBM	Precision	0.03	(0.02, 0.03)
AdaBoost	Precision	0.07	(0.07, 0.07)
XGBoost	Precision	0.04	(0.03, 0.04)
LightBoost	Precision	0.01	(0.01, 0.01)
Logistic Regression	ROC AUC	0.03	(0.03, 0.03)
KNN	ROC AUC	0.05	(0.05, 0.05)
Random Forest	ROC AUC	0.02	(0.02, 0.02)
GBM	ROC AUC	0.02	(0.02, 0.02)
AdaBoost	ROC AUC	0.06	(0.06, 0.06)
XGBoost	ROC AUC	0.04	(0.04, 0.04)
LightGBM	ROC AUC	0.01	(0.01, 0.01)
